# Supplementary material for: mTOR-mediated cancer drug resistance suppresses autophagy and generates a druggable metabolic vulnerability
Source: Nat Commun. 2020 Sep 17;11:4684. doi: 10.1038/s41467-020-18504-7 (PMC7499183; doi:10.1038/s41467-020-18504-7)
Supplement: Supplementary file 1 — Supplementary Information [file 41467_2020_18504_MOESM1_ESM.pdf]

mTOR-mediated cancer drug resistance suppresses autophagy and generates a druggable metabolic vulnerability

Gremke et al.

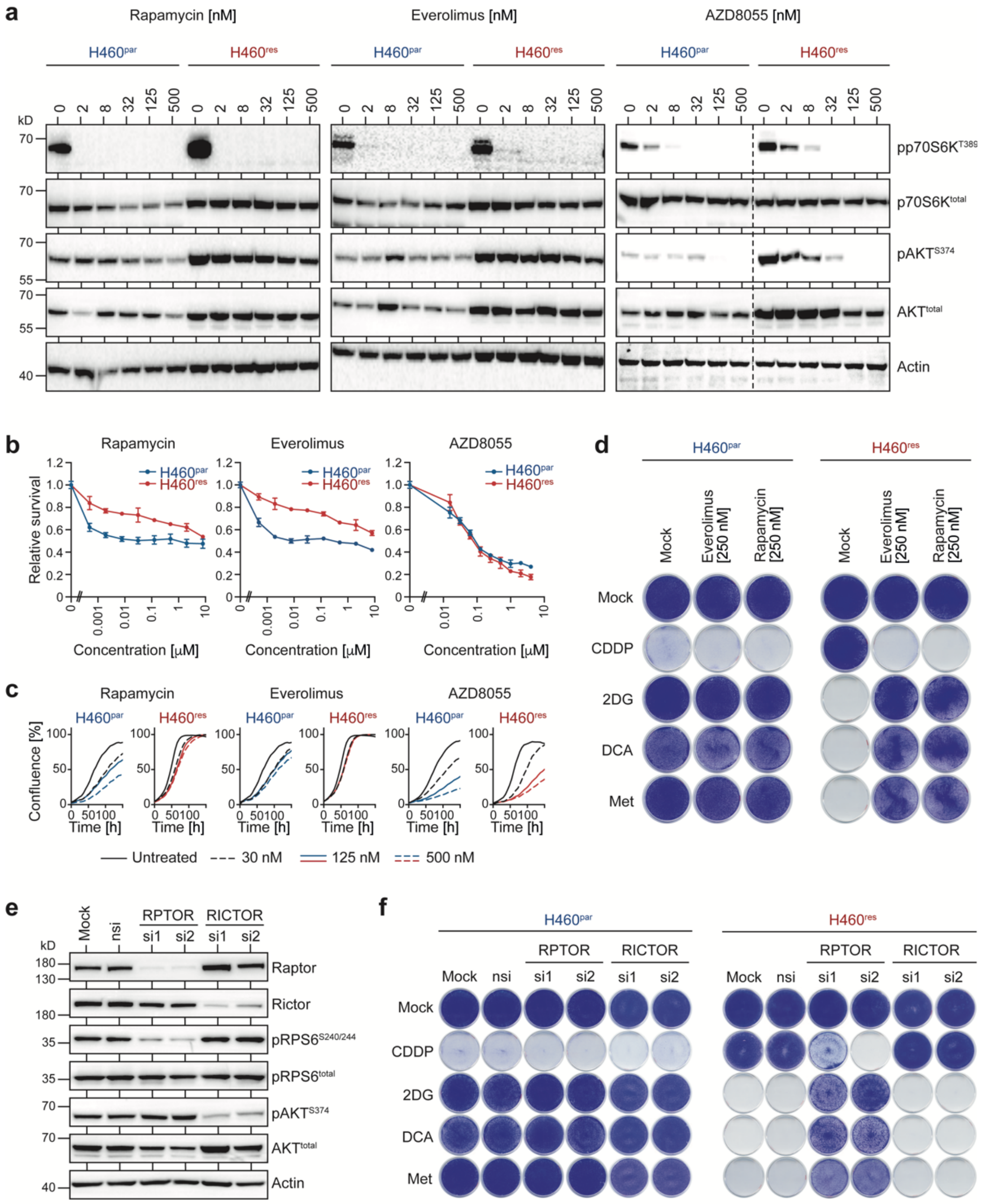

Supplementary Fig. 1. Metabolic vulnerability of CDDP-resistant H460 cells is mediated by mTORC1. a-**b**, Western blot (**a**) and cell titer assay (**b**) of H460^{par} and H460^{res} cells treated for 3 days with indicated concentrations of the mTORC1-selective inhibitors Rapamycin and Everolimus or the dual mTOR kinase inhibitor AZD8055. Shown are mean ± SD (n=3). **c**, Culture confluence determined by automated real-time live-cell imaging of H460^{par} and H460^{res} cells treated as in (**a-b**). **d**, Clonogenic growth of H460^{par} and H460^{res} cells treated with CDDP, 2DG, DCA and Metformin (Met). mTORC1 was inhibited with Rapamycin (250 nM) and Everolimus (250 nM) as indicated. **e**, Western blot of H460^{par} and H460^{res} cells 3 days following transfection with indicated siRNAs against RPTOR (Raptor) or RICTOR. **f**, Clonogenic growth of H460^{par} and H460^{res} cells transfected with indicated siRNAs 2 days before treatment with CDDP, 2DG, DCA and Met.

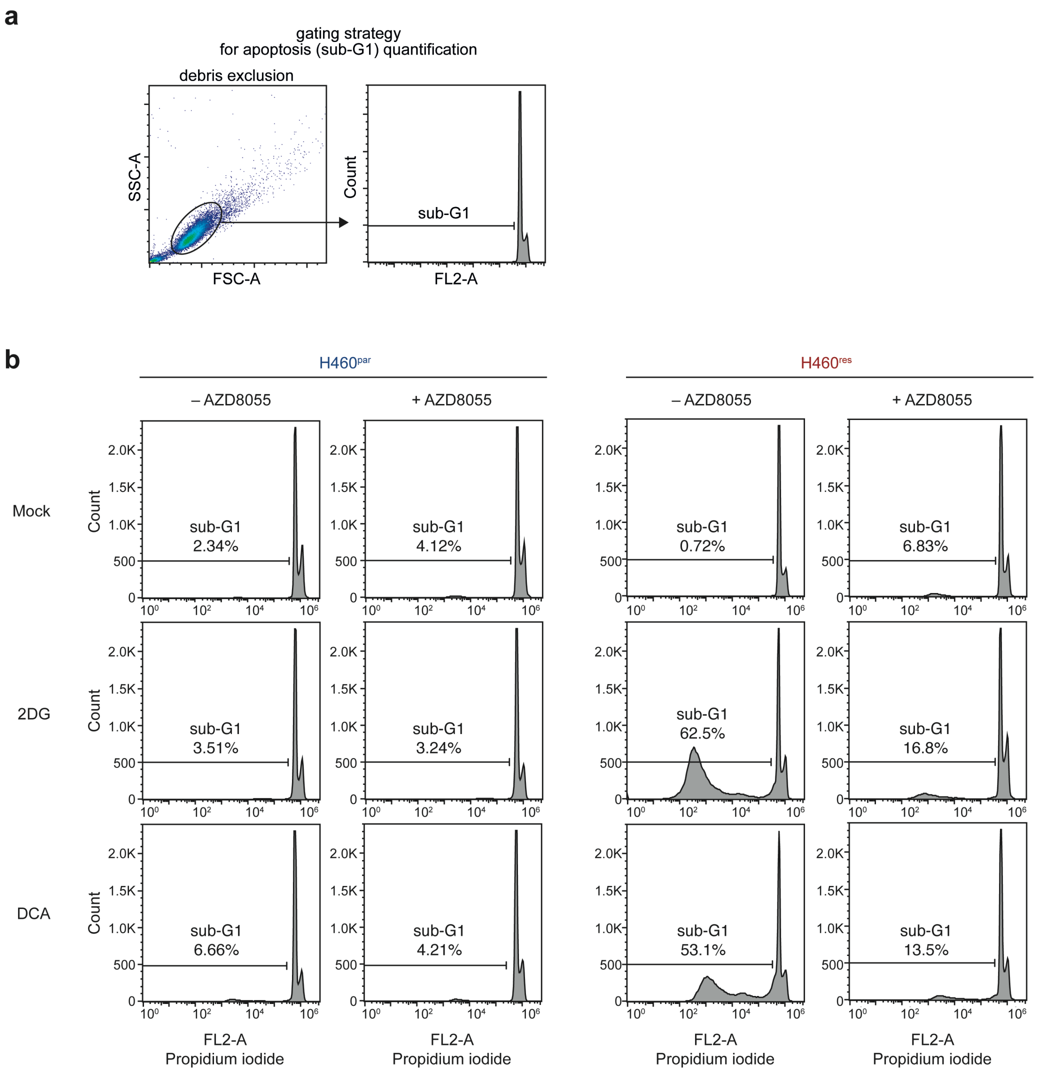

Supplementary Fig. 2. mTOR inhibition rescues CDDP-resistant H460^res cells from apoptosis induced by metabolic drug treatment. H460^par and H460^res cells were treated with the 2DG/DCA ± AZD8055 for 5 days and analyzed for apoptosis (sub-G1) by flow cytometry. **a**, Gating strategy. **b**, Representative flow cytometry histograms. The full analysis results are shown in Fig. 2e.

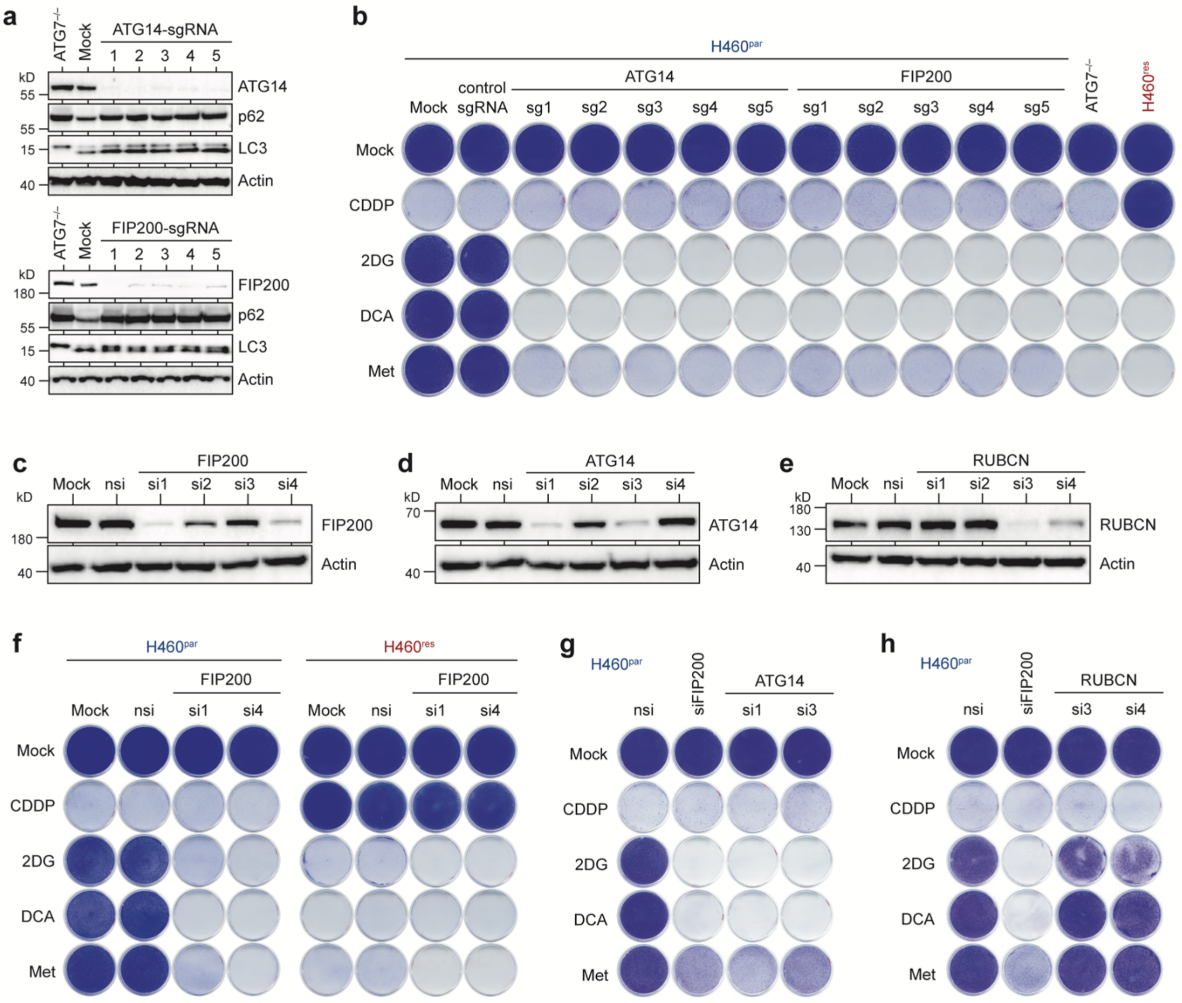

Supplementary Fig. 3. Specific inhibition of autophagy induces metabolic vulnerability. a-b, H460^{par} cells were transfected with pX459 plasmid expressing distinct CRISPR-Cas9 nucleases targeting ATG14 or FIP200. Transfected cells were selected with puromycin and examined for target gene knockout by Western blot (**a**). **b,** Clonogenic growth of knockout cells in the presence of indicated drugs. A non-targeting control nuclease was used as negative control. Positive controls were H460 ATG7$^{-/-}$ and H460^{res} cells. **c-h**, H460^{par} and H460^{res} cells were transfected with siRNAs targeting FIP200, ATG14 and RUBCN and examined for target gene knockdown by Western blot (**c-e**). **f-g**, Cells with successful knockdown were analyzed for clonogenic growth in the presence of the indicated drugs.

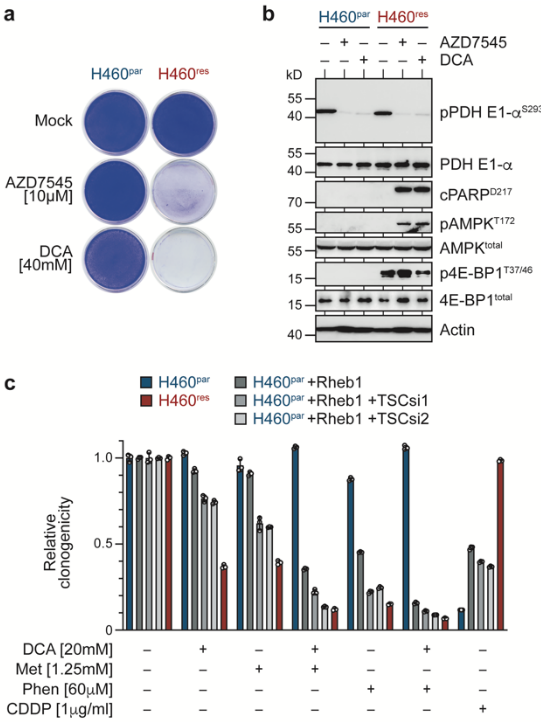

Supplementary Fig. 4. mTOR-mediated metabolic vulnerability extends to other metabolic inhibitors. a, Clonogenic growth of H460^{par} and H460^{res} cells treated with pyruvate dehydrogenase kinase inhibitors AZD7545 or DCA for 6 days. **b**, Western blot of cells H460^{par} and H460^{res} treated with AZD7545 or DCA for 48 hours. **c**, H460^{par} cells stably expressing Flag-Rheb1 were transfected with two different sets of siRNAs targeting TSC. Shown is a quantification of the clonogenic growth following treatment with indicated doses of DCA, Met, Phen and CDDP. H460^{par} and H460^{res} were included as control. Relative clonogenicity is shown as mean ± SD, n=3.

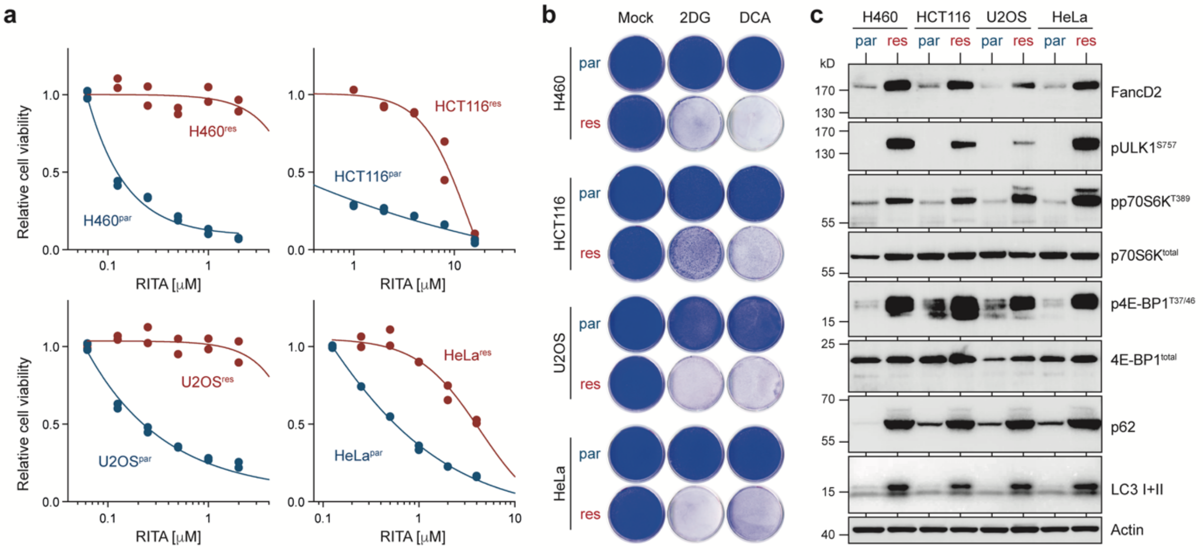

Supplementary Fig. 5. Metabolic vulnerability of RITA-resistant tumor cells. H460 lung, HCT116 colorectal, U2OS osteosarcoma and HeLa cervical cancer cells were cultured in the presence of RITA yielding RITA-resistant (res) cell lines. **a**, Cell viability following 3 day treatment with indicated RITA concentrations. Shown are individual replicates, n=2. **b**, Clonogenic growth under treatment with 2DG or DCA. H460: 20 mM 2DG, 30 mM DCA; HCT116; 15 mM 2DG, 30 mM DCA; U2OS: 20 mM 2-DG, 35 mM DCA; Hela: 12.5 mM 2DG, and 20 mM DCA. **c**, Western blot for FancD2, mTOR and autophagy markers.

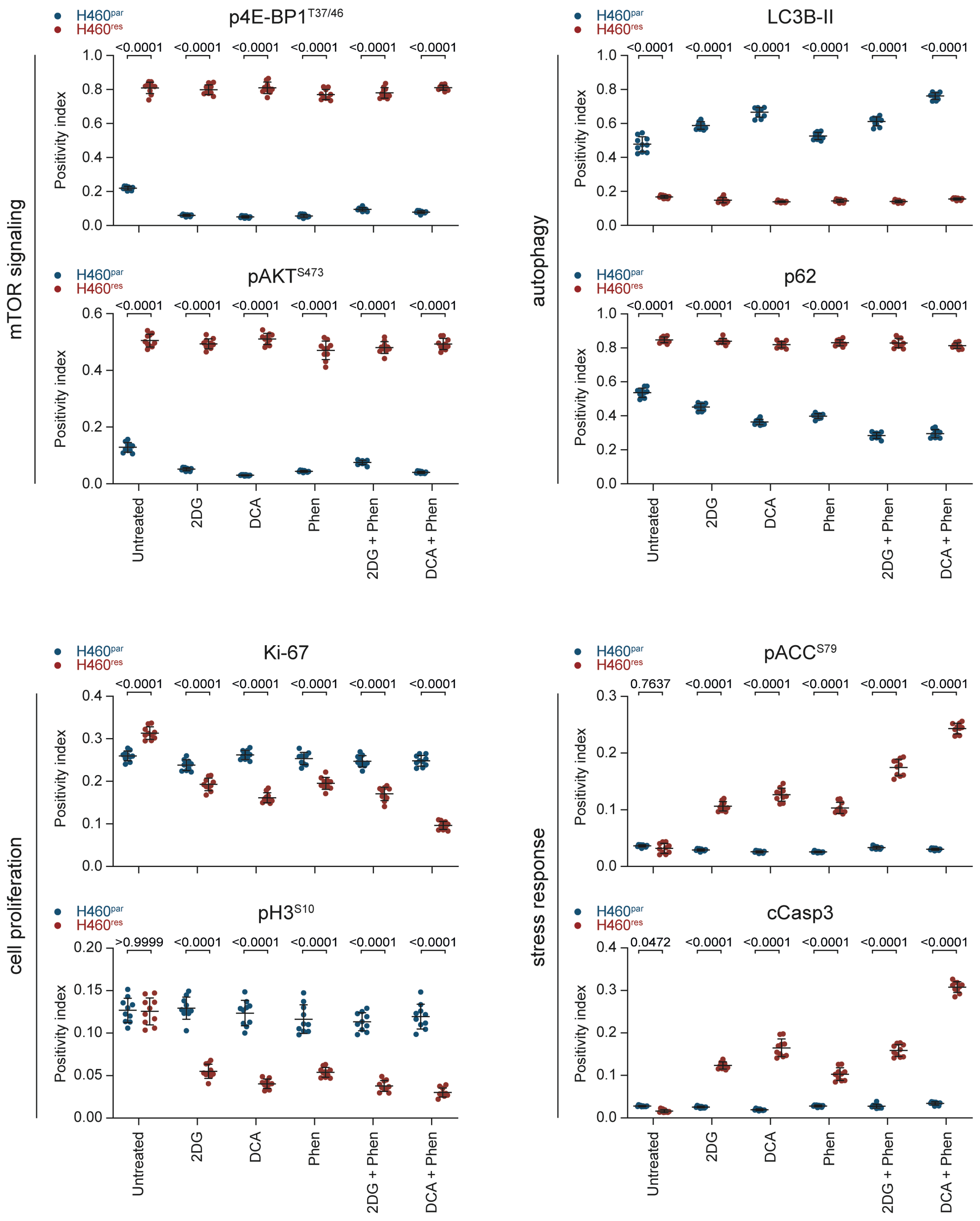

Supplementary Fig. 6. Differential effects of metabolic inhibitors on H460^{par} and H460^{res} tumors in mice. H460^{par} and H460^{res} mouse tumors treated with the indicated drugs for 3 days were examined by immunohistochemistry. Immunostaining was analyzed quantitatively by automated image analysis. Shown is the positivity index (mean ± SD, n=10 lung tumors per treatment). Differences between H460^{par} and H460^{res} mouse tumors were tested for statistical significance by two-way ANOVA and Sidak’s multiple comparisons test. Representative images are shown in Fig. 7c.
