# Supplementary material for: mTOR-mediated cancer drug resistance suppresses autophagy and generates a druggable metabolic vulnerability
Source: Nat Commun. 2020 Sep 17;11:4684. doi: 10.1038/s41467-020-18504-7 (PMC7499183; doi:10.1038/s41467-020-18504-7)
Supplement: Supplementary file 3 — Reporting Summary [file 41467_2020_18504_MOESM3_ESM.pdf]

nature research

Corresponding author(s): Thorsten Stiewe

Last updated by author(s): Aug 20, 2020

Reporting Summary

Nature Research wishes to improve the reproducibility of the work that we publish. This form provides structure for consistency and transparency in reporting. For further information on Nature Research policies, see our Editorial Policies and the Editorial Policy Checklist.

Statistics

For all statistical analyses, confirm that the following items are present in the figure legend, table legend, main text, or Methods section.

n/a	Confirmed	
☐	☒	The exact sample size (*n*) for each experimental group/condition, given as a discrete number and unit of measurement
☐	☒	A statement on whether measurements were taken from distinct samples or whether the same sample was measured repeatedly
☐	☒	The statistical test(s) used AND whether they are one- or two-sided<br>*Only common tests should be described solely by name; describe more complex techniques in the Methods section.*
☐	☒	A description of all covariates tested
☐	☒	A description of any assumptions or corrections, such as tests of normality and adjustment for multiple comparisons
☐	☒	A full description of the statistical parameters including central tendency (e.g. means) or other basic estimates (e.g. regression coefficient) AND variation (e.g. standard deviation) or associated estimates of uncertainty (e.g. confidence intervals)
☐	☒	For null hypothesis testing, the test statistic (e.g. *F*, *t*, *r*) with confidence intervals, effect sizes, degrees of freedom and *P* value noted<br>*Give P values as exact values whenever suitable.*
☒	☐	For Bayesian analysis, information on the choice of priors and Markov chain Monte Carlo settings
☒	☐	For hierarchical and complex designs, identification of the appropriate level for tests and full reporting of outcomes
☒	☐	Estimates of effect sizes (e.g. Cohen's *d*, Pearson's *r*), indicating how they were calculated

Our web collection on statistics for biologists contains articles on many of the points above.

Software and code

Policy information about availability of computer code

Data collection	Live cell imaging: samples were run on an IncuCyte S3 Live-Cell Analysis System (Sartorius) using the IncuCyte S3 2018A software<br>Flow cytometry: samples were run on an Accuri C6 Plus Cytometer (Software version 264.21, BD Bioscience)<br>Immunofluorescence microscopy: images were obtained on the Leica DM 5500 B microscope using the Leica Aperio Software (Aperio Versa v. 1.0.3.37, Aperio ImageScope v 12.3.2.8013)<br>Immunohistochemistry: images were obtained using the Ariol DM6000 B Workstation (Leica)
Data analysis	Statistics: analyses were performed using Prism software (version 8.1-8.4, GraphPad Software, USA) and Microsoft Excel for Mac (v. 16.29)<br>Flow cytometry: data were analyzed using the Accuri C6 Plus Cytometer Software version 264.21 (BD Bioscience)<br>Immunofluorescence: images were analyzed for LC3-GFP puncta in LAS X Software (Leica)<br>Immunohistochemistry: images were analyzed with the Aperio eSlide Manager Software, Version 12.3.2.5030 (Leica)<br>Live cell imaging: data were analyzed using the IncuCyte S3 Live-Cell Analysis System (Sartorius) running the IncuCyte S3 2018A software

For manuscripts utilizing custom algorithms or software that are central to the research but not yet described in published literature, software must be made available to editors and reviewers. We strongly encourage code deposition in a community repository (e.g. GitHub). See the Nature Research guidelines for submitting code & software for further information.

Data

Policy information about availability of data

All manuscripts must include a data availability statement. This statement should provide the following information, where applicable:

- Accession codes, unique identifiers, or web links for publicly available datasets
- A list of figures that have associated raw data
- A description of any restrictions on data availability

All data generated or analysed during this study are included in this published article (and its supplementary information files). Source data: unprocessed images are provided for Figures 1b, 2a, 2b, 2c, 2f, 2h, 2j, 4b, 5b, 5d, 6b, 7b, Supplementary Figures 1, 3, 4 and 5. Numerical source data are provided for Figures 1c, 1e, 1g, 1h, 2e, 2g, 2k, 3a, 3b, 3c, 3e, 4f, 4g, 5a, 5c, 6a, 6c, 6d, 6e, 7a, 7d, Supplementary Figures 1, 4, 5 and 6.

Field-specific reporting

Please select the one below that is the best fit for your research. If you are not sure, read the appropriate sections before making your selection.

☒ Life sciences ☐ Behavioural & social sciences ☐ Ecological, evolutionary & environmental sciences

For a reference copy of the document with all sections, see nature.com/documents/nr-reporting-summary-flat.pdf

Life sciences study design

All studies must disclose on these points even when the disclosure is negative.

Sample size	Sample sizes were estimated based on previous experiments conducted in our laboratory, providing sufficient numbers of replicates in each experimental group to yield a two-sided statistical test with the potential to reject the null hypothesis with a power of (1-beta) of 80% and alpha = 0.05.
Data exclusions	Fig. 7a: Animals that failed to develop tumours (3 in the CDDP and 2 in the Vehicle cohort) were excluded from the analysis.<br>Fig. 7d: Animals that reached humane endpoints before the planned end of the experiment (2 in the H460par, 4 in the H460res cohort) or failed to develop tumours (2 in the H460par, 6 in the H460res cohort) were excluded from the analysis.
Replication	Cell culture experiments were replicated in at least two independent experiments with similar results. Only results depicted in Supplementary Fig. 3a-b were performed as a single experiment, as the conclusions were validated by the RNAi experiments depicted in (Supplementary Fig. 3c, d, f, g). In addition, all experiments with H460res cells were reproduced with an independently generated CDDP-resistant H460 cell clone which yielded similar results.<br>Animal experiments were performed only once because of animal welfare reasons (3R's). Instead, a priori group size estimation was used to obtain sufficient power and significance to draw reliable conclusions from a single experiment.
Randomization	Animal experiments: For the experiment depicted in Fig. 4a and b, animals were injected with a mixture of test (H460res) and control (H460par) tumour cells, so that both cell types could be directly compared within one animal. For the experiment depicted in Fig. 4c and d, tumour cells were injected intravenously to seed multiple small lung tumor nodules that are not visible from the outside and therefore do not influence allocation to treatment groups. The experimental design therefore obviated the need for randomization. In all experiments, animals were allocated to treatment groups before visible signs of tumour development to avoid a source of bias.<br>Randomization was not relevant for other experiments, as they were performed in cell lines.
Blinding	Animal experiments: Investigators were not blinded, because the experimental setup ensured that both cell types were either treated equally (Fig. 4a and b) or tumour growth was not visible during mouse handling (Fig. 4c and d). Immunohistochemistry of mouse tumours was performed blinded by automated staining and automated image analysis.<br>Cell culture experiments: no blinding, as the same investigator performed most experiments and analyzed the data.

Reporting for specific materials, systems and methods

We require information from authors about some types of materials, experimental systems and methods used in many studies. Here, indicate whether each material, system or method listed is relevant to your study. If you are not sure if a list item applies to your research, read the appropriate section before selecting a response.

Materials & experimental systems

n/a	Involved in the study	
☐	☒	Antibodies
☐	☒	Eukaryotic cell lines
☒	☐	Palaeontology and archaeology
☐	☒	Animals and other organisms
☒	☐	Human research participants
☒	☐	Clinical data
☒	☐	Dual use research of concern

Methods

n/a	Involved in the study	
☒	☐	ChIP-seq
☐	☒	Flow cytometry
☒	☐	MRI-based neuroimaging

Antibodies

Antibodies used

ANTIBODY_COMPANY_CATALOGUE No_CLONAL_(CLONE)_DILUTION (Technique)
cleaved PARP (Asp214)_Cell Signaling_#9541_Rabbit polyclonal_1:1000 (WB)
FANCD2_Santa Cruz_sc-20022_Mouse monoclonal (FI17)_1:500 (WB)
FLAG-tag_Sigma-Aldrich_F1804_Mouse monoclonal (M2)_1:1000 (WB)
mTOR_Cell Signaling_#2983_Rabbit monoclonal (7C10)_1:1000 (WB)
Hamartin/TSC1_Cell Signaling_#6935_Rabbit monoclonal (D43E2)_1:1000 (WB)
Tuberin/TSC2_Cell Signaling_#4308_Rabbit monoclonal (D93F12)_1:1000 (WB)
Raptor_Cell Signaling_#2280_Rabbit monoclonal (24C12)_1:1000 (WB)
Rictor_Cell Signaling_#2114_Rabbit monoclonal (53A2)_1:1000 (WB)
phospho-p70S6Kinase (Thr389)_Cell Signaling_#9234_Rabbit monoclonal (108D2)_1:1000 (WB)
p70S6Kinase_Santa Cruz_sc-8418_Mouse monoclonal (H9)_1:500 (WB)
phospho-4E-BP1 (Thr37/46)_Cell Signaling_#2855_Rabbit monoclonal (236B4)_1:1000 (WB)
4E-BP1_Santa Cruz_sc-6936_Rabbit polyclonal (R-113)_1:200 (WB)
phospho-S6 Ribosomal protein (Ser240/244)_Cell Signaling_#2215_Rabbit polyclonal_1:1000 (WB)
S6 Ribosomal protein_Cell Signaling_#2217_Rabbit monoclonal (5G10)_1:1000 (WB)
phospho-AKT Ser473_Cell Signaling_#4060_Rabbit monoclonal (D9E)_1:1000 (WB)
AKT_Cell Signaling_#9272_Rabbit polyclonal_1:1000 (WB)
phospho-AMPK (Thr172)_Cell Signaling_#2535_Rabbit monoclonal (40H9)_1:1000 (WB)
AMPKalpha_Cell Signaling_#2603_Rabbit monoclonal (23A3)_1:1000 (WB)
phospho-Acetyl-CoA Carboxylase (Ser79)_Cell Signaling_#3661_Rabbit polyclonal_1:1000 (WB)
Acetyl-CoA Carboxylase_Cell Signaling_#3676_Rabbit monoclonal_1:1000 (WB)
phospho-Pyruvate Dehydrogenase E1-alpha subunit (S293)_Abcam_ab92696_Rabbit polyclonal_1:500 (WB)
Pyruvate Dehydrogenase E1-alpha subunit_Abcam_ab110330_Mouse monoclonal (9H9AF5)_1:500 (WB)
phospho-ULK1 (Ser757)_Cell Signaling_#14202_Rabbit monoclonal (D7O6U)_1:1000 (WB)
ULK1 Cell Signaling #4773_Rabbit monoclonal (R600)_1:1000 (WB)
p62/SQSTM1_Sigma-Aldrich_P0067_Rabbit polyclonal_1:1000 (WB)
LC3B (LC3I/II)_Abcam_ab48394_Rabbit polyclonal_1:1000 (WB)
ATG7_Cell Signaling_#8558_Rabbit monoclonal (D12B11)_1:1000 (WB)
ATG14_Cell Signaling_#5504_Rabbit monoclonal_1:1000 (WB)
FIP200_Cell Signaling_#12436_Rabbit monoclonal (D10D11)_1:1000 (WB)
RUBCN_Cell Signaling_#8465_Rabbit monoclonal (D9F7)_1:1000 (WB)
Gaussia Luciferase (GLuc)_Nanolight_#401P_Rabbit polyclonal (RRID:AB_2572411)_1:1000 (WB)
Cypridina Luciferase (CLuc)_antikoerper-online.de_ABIN1605705_Rabbit polyclonal (AA1-168)_1:200 (WB)
Actin_Abcam_ab6276_Mouse monoclonal (AC-15)_1:10.000 (WB)
Cleaved Caspase-3 Asp175_Cell Signaling_#9664_Rabbit monoclonal (5A1E)_1:200 (IHC)
phospho-Acetyl-CoA-Carboxylase Ser79_Cell Signaling_#3661_Rabbit polyclonal_1:500 (IHC)
phospho-Histone H3 Ser10_Cell Signaling_#9701_Rabbit polyclonal_1:200 (IHC)
Ki67_Abcam_ab15580_Rabbit polyclonal_1:75 (IHC)
phospho-4E-BP1 Thr37/46_Cell Signaling_#2855_Rabbit monoclonal (236B4)_1:1000 IHC)
phospho-AKT Ser473_Cell Signaling_#4060_Rabbit monoclonal (D9E)_1:25 (IHC)
p62/SQSTM1_Abcam_ab56416_Mouse monoclonal_1:20,000 (IHC)
LC3B-II_Abcam_ab48394_Rabbit polyclonal_1:2000 (IHC)
anti-mouse IgG-HRP from sheep: Cytiva (formerly GE Healthcare)_#NA9310_1:5000 (WB)
anti-mouse IgG-HRP from goat: Thermo Fisher Scientific_#A16084_1:5000 (WB)
anti-rabbit IgG-HRP from donkey: Cytiva (formerly GE Healthcare)_#NA9340_1:5000 (WB)

Validation

All antibodies have been validated by the manufacturer. We only used antibodies recommended by the manufacturer for the species and application mentioned above.

Antibodies by Cell Signaling: "At Cell Signaling Technology (CST), we understand that there is no single assay that can determine the validity of an antibody. Confirming that an immunoreagent is sufficiently s pecific and sensitive depends on the application and protocol being used, the type and quality of sample being analyzed, and the inherent biophysical properties of the antibody itself. To ensure our antibodies will work in your experiment, we adhere to the Hallmarks of Antibody ValidationTM, six complementary

strategies that can be used to determine the functionality, specificity, and sensitivity of an antibody in any given assay. CST adapted the work by Uhlen, et. al., ("A Proposal for Validation of Antibodies." Nature Methods (2016)) to build the Hallmarks of Antibody Validation, based on our decades of experience as an antibody manufacturer and our dedication to reproducible science."

Antibodies by Abcam: "Antibodies are validated in western blot using lysates from cells or tissues that we have identified to express the protein of interest. Once we have determined the right lysates to use, western blots are run and the band size is checked for the expected molecular weight. We will always run several controls in the same western blot experiment, including positive lysate and negative lysate. When possible, we also include knock-out (KO) cell lines as a true negative control for our western blots. We are always increasing the number of KO-validated antibodies we provide. In addition, we run old stock alongside our new stock. If we know the old stock works well, this also acts as a suitable positive control.If the western blot result gives a clear clean band and we are happy with the result from the control lanes, these antibodies will be passed and added to the catalog."

Eukaryotic cell lines

Policy information about cell lines

Cell line source(s)	HCT116, U2OS, Hela, T47D, H460, H1975 (ATCC)<br>KrasG12D/wt;Trp53-/- lung adenocarcinoma cells were obtained from lung tumours arising in Adeno-Cre infected KrasLSLG12D/wt;Trp53flox/flox mice
Authentication	STR profiling (DSMZ Braunschweig)
Mycoplasma contamination	All cell lines were tested monthly for mycoplasma. Only mycoplasma-negative cell lines were used for experiments.
Commonly misidentified lines<br>(See ICLAC register)	none

Animals and other organisms

Policy information about studies involving animals; ARRIVE guidelines recommended for reporting animal research

Laboratory animals	Mus musculus: C;129S4-Rag2tm1.1Flv;Il2rgtm1.1Flv /JThst, 6-12 week-old, male and female. All mice were bred and maintained under specified-pathogen-free conditions at a room temperature of 22±1°, a relative humidity of 50 ±10% and a 12/12 dark/light cycle. Mice were kept in groups of 3-7 and provided continuously with sterile water and chow pellets.
Wild animals	none
Field-collected samples	none
Ethics oversight	Dezernat 54 Veterinärwesen und Verbraucherschutz, Regierungspräsidium Giessen, Germany

Note that full information on the approval of the study protocol must also be provided in the manuscript.

Flow Cytometry

Plots

Confirm that:

- [x] The axis labels state the marker and fluorochrome used (e.g. CD4-FITC).
- [x] The axis scales are clearly visible. Include numbers along axes only for bottom left plot of group (a 'group' is an analysis of identical markers).
- [x] All plots are contour plots with outliers or pseudocolor plots.
- [x] A numerical value for number of cells or percentage (with statistics) is provided.

Methodology

Sample preparation	Adherent cells were trypsinised, combined with cell culture supernatant and washed in 5 ml PBS. Cell pellet was resuspended in 1 ml PBS and fixed by drop-wise addition of 10 ml ice-cold 90% ethanol while vortexing. After fixation overnight, cells were stained with 10 µg ml-1 propidium iodide supplemented with 100 µg ml-1 RNase A. Cells were analysed for sub-G1 by flow cytometry.
Instrument	Accuri C6 Plus cytometer (BD Bioscience).
Software	BD AccuriTM C6 Software Version 264.21
Cell population abundance	No sorting was performed.

Gating strategy	Tumour cells were identified in untreated cell cultures based on their forward scatter (FSC-A) and side scatter (SSC-A) profiles and a FSC/SSC-gate was used to exclude cell debris with a small FSC. The same gate was applied to all treated samples. An example is provided in Supplementary Figure 2a.

☒ Tick this box to confirm that a figure exemplifying the gating strategy is provided in the Supplementary Information.
